# The Use of Ammonia and Strychnine in the Treatment of Pneumonia

**Published:** 1881-01

**Authors:** 


The use of Ammonia and Strychnine in the Treatment of Pneumonia.—Dr. John Dell' Orto, advocates, and reports his experience with these articles in a lengthy paper in the *New Orleans Medical Journal* for November. He uses Fothergill's prescription, viz: *Five grains* of carbonate of ammonia, and *fifteen drops* of tincture of nux-vomica, with a little water and syrup, to be taken *three* times a day.

